# Neuropsychiatric phenotype in relation to gene variants in the hemizygous allele in 3q29 deletion carriers: A case series

**DOI:** 10.1002/mgg3.889

**Published:** 2019-07-25

**Authors:** Eva Albertsen Malt, Katalin Juhasz, Anna Frengen, Teresia Wangensteen, Nina Merete Emilsen, Borre Hansen, Oleg Agafonov, Hilde Loge Nilsen

**Affiliations:** ^1^ Department of Adult Habilitation Akershus University Hospital Lorenskog Norway; ^2^ Campus Ahus, Institute of Clinical Medicine University of Oslo Oslo Norway; ^3^ Section for Clinical Molecular Biology Akershus University Hospital Lorenskog Norway; ^4^ Department of Medical Genetics Oslo University Hospital Oslo Norway; ^5^ Bioinformatics Core Facility, Department of Core Facilities, Institute of Cancer Research Radium Hospital, Part of Oslo University Hospital Oslo Norway

**Keywords:** 3q29 deletion, autistic disorder, cilia, schizophrenia, synaptic function

## Abstract

**Background:**

Genetic risk variants in the hemizygous allele may influence neuropsychiatric manifestations and clinical course in 3q29 deletion carriers.

**Methods:**

In‐depth phenotypic assessment in two deletion carriers included medical records, medical, genetic, psychiatric and neuropsychological evaluations, brain MRI scan and EEG. Blood samples were analyzed for copy number variations, and deep sequencing of the affected 3q29 region was performed in patients and seven first‐degree relatives. Risk variants were identified through bioinformatic analysis.

**Results:**

One deletion carrier was diagnosed with learning difficulties and childhood autism, the other with mild intellectual disability and schizophrenia. EEG abnormalities in childhood normalized in adulthood in both. Cognitive abilities improved during adolescence in one deletion carrier. Both had microcytic, hypochromic erythrocytes and suffered from chronic pain and fatigue. Molecular and bioinformatic analyses identified risk variants in the hemizygous allele that were not present in the homozygous state in relatives in genes involved in cilia function and insulin action in the autistic individual and in synaptic function and neurosteroid transport in the subject with schizophrenia.

**Conclusion:**

3q29 deletion carriers may undergo developmental phenotypic transition and need regular medical follow‐up. Identified risk variants in the remaining hemizygous allele should be explored further in autism and schizophrenia research.

## 1 INTRODUCTION

Copy number variation (CNV), which involves unbalanced rearrangements that lead to gain (duplication) or loss (deletion) of longer DNA fragments, is a common form of genetic variation in the human genome. CNV presents as a continuous spectrum of phenotypes in the population, ranging from polymorphisms to severe Mendelian conditions (Zarrei, MacDonald, Merico, & Scherer, 2015). Many CNVs are associated with neurodevelopmental and neuropsychiatric disorders (Davis et al., 2009). However, it has become evident that CNVs do not have high diagnostic specificity and a given CNV can give rise to many different neurodevelopmental and neuropsychiatric phenotypes, including bipolar disorder, schizophrenia, autism spectrum disorder (ASD), intellectual disability (ID) or developmental delay (DD), attention deficit hyperactivity disorder (ADHD), and epilepsy (Grayton, Fernandes, Rujescu, & Collier, 2012; Kirov, 2015; Torres, Barbosa, & Maciel, 2016). In deletion carriers, haploinsufficiency of critical dose‐dependent genes may account for some of the syndromic features associated with the specific CNV, but this alone cannot explain the neuropsychiatric pleiotropy (Chenier et al., 2014). It is now of great importance to understand the contributions of the individual genes and gene variants within a CNV region to allow us to understand their role in deletions and reciprocal duplications.

Pleiotropy of the neuropsychiatric phenotype also holds for the approximately 1.5‐Mb recurrent deletion in the subtelomeric region of the long arm on chromosome 3 (3q29), which was first described in 2005 in six patients with ID/DD and minor facial dysmorphisms (OMIM 609,424) (Willatt et al., 2005). To date, a total of 44 persons with similar CNVs have been reported in the literature (Glassford, Rosenfeld, Freedman, Zwick, & Mulle, 2016). Variable structural abnormalities and ID/DD have been found in almost all cases (Glassford et al., 2016; Quintero‐Rivera, Sharifi‐Hannauer, & Martinez‐Agosto, 2010; Willatt et al., 2005). The neurodevelopmental/neuropsychiatric phenotypic spectrum includes language disorder, ASD, ADHD of the inattentive type, severe depression, bipolar disorder, childhood psychosis, and schizophrenia (Citta et al., 2013; Clayton‐Smith, Giblin, Smith, Dunn, & Willatt, 2010; Cox & Butler, 2015; Mulle et al., 2010; Quintero‐Rivera et al., 2010; Sagar et al., 2013; Willatt et al., 2005). A meta‐analysis concluded that the CNV was associated with a 40‐fold increased risk for schizophrenia, indicating that the 3q29 deletion may be the single largest risk factor for schizophrenia, exceeding even the 22q11.2 deletion (Mulle, 2015).

The region affected by the 3q29 CNV region contains 21 protein‐coding genes. Haploinsufficiency of *FBXO45* (OMIM 609,112), *DLG1* (OMIM 601,014), and *PAK2* (OMIM 605,022) have been proposed as causative of the neuropsychiatric manifestations because they play putative roles in synaptic transmission (Carroll et al., 2011). Genes with impact on cilia function (*CEP19* (OMIM 615,586), *TCTEX1D2* (OMIM 617,353) (Marley & von Zastrow, 2012; Youn & Han, 2018), neurosteroid transport (*SLC51A* (OMIM 612,084)) (Cai, Cao, Zhou, & Yao, 2018; Grube, Hagen, & Jedlitschky, 2018; Tuem & Atey, 2017), iron homeostasis, and synaptic plasticity (*TFRC* (OMIM 190,010)) (Liu et al., 2016; Matak et al., 2016) are also of great interest regarding neuropsychiatric diversity. In addition to the possible effects of haploinsufficiency, another hypothesis lies in the fact that genetic variation within the nondeleted 3q29 allele could influence the neuropsychiatric phenotype. This has been explored previously in two studies regarding the 22q11.2 deletion syndrome, but never in 3q29 deletion carriers (Gothelf et al., 2005; Guipponi et al., 2017).

Only longitudinal follow‐up of the patients can help to elucidate the developmental trajectory of a certain CNV. We need to consider that the neuropsychiatric phenotype might undergo phenotypic transition during development, as has been documented for patients with the 22q11.2 deletion syndrome (Swillen & McDonald‐McGinn, 2015). Regarding 3q29 deletions, a systematic neuropsychiatric evaluation with special emphasis on cognitive and behavioral assessment has only been published in one case report of four children by Citta et al. (2013). In adults, only self‐report data from five patients are included in the 3q29 Deletion Registry (Glassford et al., 2016), and little is known about developmental phenotypic transition and the adult phenotype. Therefore, systematic clinical in‐depth characterization of adult carriers of the 3q29 deletion is needed. The estimated prevalence of the deletion is 1:30,000 in the general population, which makes it necessary to look critically for feasible approaches to study phenotypic pleiotropy in this rare disorder.

Family studies are fundamental tools in the discipline of behavioral genetics, as they permit assessments of degrees of familial resemblance, or aggregation of physical, psychological, and behavioral characteristics. Adoption studies and comparisons between monozygotic and dizygotic twins are the gold standard of these studies. In studies of rare behavioral and pleiotropic phenotypes, a supplementary approach could be to study genetic variants and phenotypes in a context that minimizes as much as possible the impact of general genetic background and environmental influences. This minimization can be achieved by studying CNV carriers together with their first‐degree relatives. Such an approach could allow the generation of well‐founded hypotheses about genotype‐phenotype associations, which could later be tested in larger patient samples.

In this study, we want to explore the hypothesis that genetic variation in the remaining hemizygous allele contributes to phenotype variability in 3q29 deletion carriers. We have therefore performed in‐depth phenotyping of two patients with 3q29 deletion syndrome and targeted deep sequencing of the affected 3q29 region in patients and first‐degree relatives.

## 2 MATERIALS AND METHODS

### 2.1 Ethical compliance

The study adhered to the tenets of the Declaration of Helsinki. The Regional Ethical Committee of South‐Eastern Norway approved the study, and the participants or their legal representatives provided written and oral informed consent. All participants were informed that they could withdraw from the study at any time without any negative consequences. The participants and their legal guardian gave their consent for publication.

### 2.2 Patients

Two consecutive families with one first‐degree family member diagnosed with 3q29 deletions were included in the study. Family 1 was of European ethnicity, and patient 1 was a 23‐year‐old male. Family 2 was of South Central Asian ethnicity, and patient 2 was a 29‐year‐old female. Informed consent was obtained from all participants.

### 2.3 Clinical and phenotypic assessments

Clinical information was collected from patients and first‐degree relatives by experienced staff, including a psychiatrist, a neuropsychologist, and clinical geneticists. For patients, all available lifetime medical records were collected, and a thorough developmental and general anamnestic history was taken. At inclusion, routine physical and neurological examinations were performed. Dysmorphic features and congenital anomalies were evaluated. ICD‐10 diagnoses were assessed through a semi‐structured neuropsychiatric interview, The M.I.N.I. Plus International Neuropsychiatric Interview (Norwegian Translation Version 6.0.0) (Leiknes, Malt, Malt, & Leganger, 2006), and a clinical interview with a parent. ASD was assessed at age 5 years 8 months in a psychiatric unit specialized in assessment of neurodevelopmental disorders in children. Assessments included the Autism Diagnostic Interview‐Revised (ADI‐R) (Lord, Rutter, & Couteur, 1994), the Autism Diagnostic Observation Schedule (ADOS), module 2 and 3 (Lord et al., 1989) and the ADI‐R and Vineland Adaptive Behavior Scales (Sparrow, Balla, & Cicchetti, 1984). The ADOS is a diagnostic, semi‐structured clinical assessment that directly observes behaviors associated with ASD in the areas of social communication, play and interaction, and restrictive and repetitive behaviors The ADI‐R is a semi‐structured comprehensive parent/caregiver interview designed to evaluate early developmental history and current and lifetime presentation of autism symptomatology. At inclusion verbal and nonverbal cognitive abilities were assessed using the Wechsler Adult Intelligence Scale—4th edition (WAIS‐IV), the Norwegian edition with Scandinavian norms (Wechsler, Coalson, & Raiford, 2008), and Raven Matrices Practice Tests (Raven, 2000). Adaptive behavior was assessed by the semi‐structured interview Vineland Adaptive Behavior Scales, 2nd edition (VABS‐II) (Sparrow, Cicchetti, & Balla, 2005).

A brain structural magnetic resonance imaging (MRI) scan with epilepsy protocol was performed in patient 1. Data were collected on a 1.5T magnet scanner. MRI scan had been performed previously and was not repeated in patient 2. Standard EEG was performed in both patients.

Peripheral blood samples were drawn for biochemical and pharmacogenetic analyses, and a neurometabolic workup was performed after the collection of overnight fasting urine and peripheral blood samples.

First‐degree relatives underwent a brief clinical interview about psychiatric and somatic symptoms and disorders.

### 2.4 Genetic counselling

Genetic testing and counselling of patients and first‐degree relatives were conducted according to Act 2003–12–05–100 relating to human medical use of biotechnology.

### 2.5 Genomic DNA extraction and molecular analyses

In patients, peripheral venous blood was analyzed for CGG repeats using the FMR1 with extra‐long and repeated prime polymerase chain reaction. To assess genome‐wide DNA dosage imbalance, array comparative genome hybridization was performed using Agilent 180K Sureprint G3 Human CGH array in the two patients. Subsequently, the presence of the 3q29 deletion was tested in relatives by MRC Holland SALSA MLPA probemix P245 (B1‐0512). Genomic DNA from patients and first‐degree relatives was extracted from peripheral blood using the DNeasy Blood & Tissue Kit (Qiagen). DNA was sheared using a focused‐ultrasonicator (Covaris) and the fragment size of the sheared DNA was assessed by TapeStation (Agilent Technologies). An Agilent SureSelect Target Enrichment System was used to capture the genomic 3q29 region, including intergenic and intragenic regions, excluding repetitive sequences, prior to sample sequencing. The 3q29 region was sequenced by MiSeq, Illumina.

### 2.6 Bioinformatic analyses and variant classification

After base calling and removal of adaptors, raw reads were mapped to the reference genome GRCh37. Variant calling was performed using the GATK haplotype caller (v.3.7) to identify single‐nucleotide polymorphisms (SNPs) and indels (McKenna et al., 2010). Variants were filtered using the GATK recommendations. In brief, SNPs were filtered according to variant confidence (QualByDepth, QD < 2.0). Fisher's exact test (FisherStrand, FS > 60.0) and StrandOddsRatio (SOR > 3.0) were used to detect strand bias. Mapping quality across samples was assessed using RMSMappingQuality (MQ < 40.0). MappingQualityRankSumTest (MQRankSum < −12.5) was used to assess mapping qualities (reads with reference bases vs. those with the alternate allele). ReadPosRankSumTest (ReadPosRankSum <−8.0), a u‐based z‐approximation from the Mann–Whitney rank sum test, was used to assess the distance from the end of the read for reads with the alternate allele. If the alternate allele was only seen near the ends of reads, the variant was discarded. Similarly, indels were filtered using the following cutoffs: QualByDepth (QD < 2.0), FisherStrand (FS > 200.0), ReadPosRankSumTest (ReadPosRankSum < −20.0), and StrandOddsRatio (SOR > 10.0).

SnpEff v 4.3 was used to annotate variants and classify them according to a predicted impact—high, moderate, low, and modifier (Cingolani et al., 2012). A variant was assigned to have high impact in cases when it was predicted to cause protein truncation, loss of function, or trigger nonsense‐mediated decay; moderate impact when it was predicted to be nondisruptive, but possibly affecting protein function; low impact when it was predicted to be harmless; and modifier for noncoding variants or variants affecting noncoding genes where there is no evidence of impact. The variant lists were manually inspected to identify variants that segregated with the phenotype.

## 3 RESULTS

In patient 1, array‐CGH analysis identified a 1.6‐Mb deletion, Arr(hg19)3q29(195747856–197339329)x1. The deletion was paternal and de novo. In patient 2, array‐CGH identified a 1.5‐Mb deletion, Arr(hg19)3q29(195789463–197339329)x1. The mother and sisters of patient 2 did not carry the deletion, and there is no information about the father, who is deceased. In both patients, the number of CGG repeats in *FMR1* was within the normal range, and no other potentially pathogenic CNVs were detected.

### 3.1 Clinical phenotypic characteristics

Clinical characteristics of the two patients are presented in Table 1. Patient 1 was diagnosed with childhood autism (ICD‐10 F84.0), while patient 2 had early‐onset psychosis developing to paranoid schizophrenia (ICD‐10 F22.0) and was only partially responsive to treatment with antipsychotic drugs. Neither of the patients showed regression in cognitive function, and cognitive testing revealed improvement during adolescence in patient 1 with autism and stable function in patient 2 with schizophrenia. Adaptive abilities improved in patient 1 and declined in patient 2. Neither patient had epilepsy, but both patients’ EEGs were abnormal and indicated cerebral dysfunction during childhood. Both patients’ EEGs were normalized during adulthood. Both patients had hematological abnormalities persisting over time, with microcytic, hypochromic erythrocytes, and low levels of transferrin receptor. Hemoglobin was within the normal range in both patients, but serum iron was low in patient 2. Both suffered from chronic pain, especially headache, and fatigue. Patient 2 suffered from hypothyreosis and received hormonal substitution. Thyroid‐stimulating hormone was slightly elevated without elevated autoantibodies (thyroid peroxidase, thyroid receptor antibody). Other general laboratory blood tests were normal in both patients.

**Table 1:** Key developmental phenotypes in two adult carriers of the chromosome 3q29 deletion

Feature	Early childhood	Childhood–Adolescence	Adulthood
(A) Patient 1, male 23 years			
Birth/growth	Normal to term, Apgar score 9/9 length, weight and head circumference at 50 percentile Neonatal jaundice Failure to thrive, failure to gain weight	Height < 50 percentile Weight 2.5 percentile	Head circumference 25–50 percentile Height 50 percentile Underweight, BMI 15.5
Development	Described as very “calm child”	Delayed fine and gross motor, speech, and social development	
Infections, general health	Recurrent middle ear and upper respiratory tract infections	Recurrent middle ear and upper respiratory tract infections	Chronic head and back pain. Fatigue
Voice		Poor articulation, monotonous voice	Nasal voice
Musculoskeletal abnormalities		Pectus excavatum Clinodactyly second toe Gait abnormality (in‐toe gait, short steps)	Pectus excavatum (operated) Clinodactyly Kyphosis Back pain Long, slender fingers Gait abnormality
Facial dysmorphism			Broad nasal tip Upslanting palpebral fissures Slight hypertelorism Thin upper lip
Heart			ECG: Right bundle branch block Echocardiography normal
Hematology		Low hemoglobin, ferritin, and iron	Microcytosis Low transferrin receptor
EEG		Frequent spike‐slow‐wave activity in left occipital region	Normal
MRI of head		Normal	Small left side periventricular connatal cyst
Psychiatric evaluation		F84.0 Infantile autism	F84.0 Infantile autism
Cognitive evaluation		F70.0 Mild intellectual disability Low expressive verbal skills Difficulties regarding focused attention, planning, and organization Low adaptive skills	Low average IQ Difficulties regarding focused attention, planning, and organization Improved adaptive skills
Metabolic screening			Normal
(B) Patient 2, female 29 years			
Birth/growth	Normal birth at term		Short stature BMI 36 (obese) Head circumference 52 cm (2.5 percentile)
Development	Normal fine and gross motor and speech development. Delayed/reduced social development		
Infections/general health			Caries, periodontitis Sleep apnea (mild) Chronic headache and fatigue
Eyes		Exotropia, operated	
Musculoskeletal abnormalities			Clinodactyly 5th toe
Facial dysmorphism			Slightly coarse facial features Round face Low posterior hairline
Hematology		Iron deficiency anemia	Microcytosis Low transferrin receptor
Endocrine		Hypothyreosis	Hypothyreosis
EEG		Slow waves in frontotemporal regions, no epileptic activity	Normal
MRI of head		Normal	Normal
Psychiatric evaluation		Recurrent psychoses from age 14	Frequent admissions to psychiatric hospital F22.0 Paranoid schizophrenia with persisting hallucinations
Cognitive evaluation		F70.0 Mild intellectual disability	F70.0 Mild intellectual disability Stable cognitive skills, declining adaptive skills
Metabolic screening			Normal

Evaluations of first‐degree relatives of both patients indicated no psychiatric, hematological, endocrine, or other serious somatic disorders. The clinical interview, academic performance, and occupational performance indicated cognitive abilities and adaptive skills within the normal range in all first‐degree relatives.

### 3.2 Molecular and bioinformatic analyses of genetic risk variants

Identified high‐ and moderate hemizygous genetic risk variants in patients found only in the heterozygous state in first‐degree relatives are shown in Table 2. These variants were not associated with disease in any of the family members. Patient 1 carried a splice donor variant in *TCTEX1D2* predicted by the SnpEff algorithm to be a high‐risk splice variant. However, other supporting laboratory data are required in order to assess whether this variant may have functional consequences.

**Table 2:** High‐ and moderate‐risk variants in the hemizygous 3q29 allele in two deletion carriers^a^

Chr	Position	dbSNP ID/HGVS	ANN gene	ANN impact	ANN effect	P1	M1	F1	S1	B1	P2	M2	S2.1	S2.2
3	195,955,762	rs939885 NC_000003.11:g.195955762G>A	*SLC51A*	Moderate	Missense variant	0	0/0	0/1	0/0	0/1	1	0/1	0/1	0/1
3	196,019,212	rs6775861 NC_000003.11:g.196019212C>T	*TCTEX1D2*	High	Splice donor variant & intron variant	1	0/1	0/0	0/1	0/0	0	0/0	0/0	0/0
3	196,435,534	rs6776064 NC_000003.11:g.196435534T>C	*CEP19*	Moderate	Missense variant	1	0/1	0/1	0/1	0/1	1	0/1	0/1	0/1
3	196,865,242	rs1134986 NC_000003.11:g.196865242C>T	*DLG1*	Moderate	Missense variant	0	0/0	0/0	0/0	0/0	1	0/1	0/0	0/0
				Moderate	Missense variant	0	0/0	0/0	0/0	0/0	1	0/1	0/0	0/0
				Moderate	Missense variant	0	0/0	0/0	0/0	0/0	1	0/1	0/0	0/0
				Moderate	Missense variant	0	0/0	0/0	0/0	0/0	1	0/1	0/0	0/0
				High	Structural interaction variant	0	0/0	0/0	0/0	0/0	1	0/1	0/0	0/0

Note

0: Reference variant; 1: Risk variant. P1: Patient 1; Family 1. M1: Mother; Family 1. F1: Father; Family 1. S1: Sister; Family 1. B1: Brother; Family 1. P2: Patient 2; Family 2. M2: Mother; Family 2. S2.1: Sister 1; Family 2. S2.2: Sister 2; Family 2.

Only variants for which no first‐degree relatives were homozygous are presented. A variant was assigned to have high impact in cases when it was predicted to cause protein truncation, loss of function, or trigger nonsense‐mediated decay; it was assigned moderate impact when it was predicted to be nondisruptive, but possibly affecting protein function.

a hg19, GRCh37.

Patient 1 also carried a missense mutation (rs1134986) annotated by the ExAC browser to be a missense mutation in *PCYT1A*, but upon careful inspection of the validated gene predication in this region we found that this variant is not located in the *PCYT1A* gene or any other gene. Consistently, the transcript has not been experimentally verified. It cannot be excluded, however, that this variant may contributes to the phenotype of the patient through a regulatory function.

**Figure 1:**
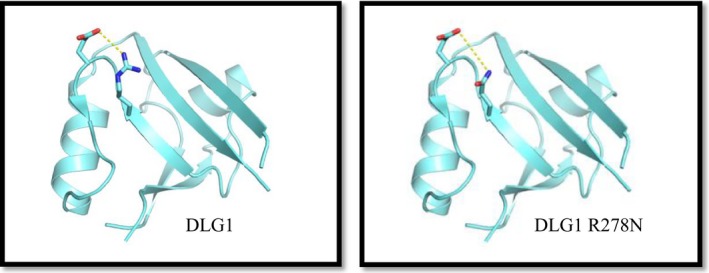
Model of DLG1 R278N mutation. Structural model showing wild type (left) and Arg278Gln (blue) mutated human DLG1 showing that the mutations induces higher degree of surface flexibility and impaired interaction with Glu281 (red)

Patient 2 carried a hemizygous a missense, structural interaction moderate‐risk variant rs1134986 in *DLG1.* This missense mutation introduces and amino acid substitution (Arg278Gln) in a conserved region of the protein. It is classified as at moderate‐risk variant as it substitutes a large and basic amino acid to a medium sized and polar residue (Carroll et al., 2011). Moreover, the variant is located in the PDZ1‐domain which mediates interaction with a range of proteins and lipids. Modeling this substitution based on a crystal structure of the human DLG1 protein (PMID:21,858,148) (Zhang et al., 2011) supports that the mutation may impair interaction with Glu281 and thereby introduce a higher degree of surface flexibility. In addition, the substitution will promote local negative surface charge that could affect interaction with protein partners (Figure 1).

Patient 2 also carried a moderate‐risk missense variant in *SLC51A,* a C to T transition corresponding to the high‐frequency polymorphism rs939885 and leading to substitution of valine for isoleucine at position 202. This variant has relatively high frequency in the normal population and the amino acids have similar chemical properties. Neither variant is reported as having known clinical significance.

Both patients shared a missense variant (rs6776064) in *CEP19*. This variant, classified as having moderate effect, leads to a Met3Val N‐terminal amino acid change in a region with no known sequence motifs or protein interaction sites.

Some moderate‐risk variants in *TFRC*, *TM4SF19*, and *PIGZ* found in the patients and *RNF168* were present in the homozygous state in first‐degree relatives. As expected, many variants classified as either as mild risk or as modifiers were identified in the patients. Similarly, intergenic variants in predicted regulatory regions that segregated with the disease phenotype were identified in both patients; however, the impact of these is currently unknown.

## 4 DISCUSSION

This is the first report that combines neurodevelopmental, in‐depth phenotypic, and genetic data to describe longitudinal phenotypic development in adult carriers of the 3q29 deletion. Both patients showed previously described traits associated with the syndrome (Cox & Butler, 2015). However, we have also identified some new features in both patients that have not been described before. These include EEG abnormalities in childhood and enduring hematological abnormalities. It is also important and worrying that both patients suffered from chronic pain and fatigue.

In line with previous observations of pleiotropy, the probands had different neuropsychiatric phenotypes (Cox & Butler, 2015). One was diagnosed with childhood autism, while the other had paranoid schizophrenia. Patient 1 was diagnosed with ID at 5 years of age, but his cognitive abilities were found to be within the normal range at 23 years of age. The impaired cognitive function in patient 2 was stable over time, although adaptive functions had declined considerably. These findings indicate that the 3q29 deletion might undergo phenotypic transition during development, as has been described previously in patients with other pathogenic CNVs. This underscores the importance of longitudinal follow‐up of 3q29 deletion carriers.

Our hypothesis was that genetic risk variants in the remaining hemizygous allele may contribute to diverse neuropsychiatric phenotypes. It is therefore interesting that the genetic analyses identified highly relevant risk variants not shared by the two patients.

Patient 1 carried a possible splice variant in *TCTEX1D2*, which codes for a ciliary protein. TCTEX1D2 is found in primary cilia and has a role in intraflagellar transport, a bidirectional microtubule‐based transport system that operates between the ciliary tip and base (Youn & Han, 2018). Primary cilia play a role in key developmental signaling pathways, and mutations that affect the structure or function of primary cilia may result in ciliopathies. The pleiotropic features of distinct ciliopathies often overlap and may include brain malformation, neurologic impairment, retinal degeneration, skeletal anomalies, congenital heart diseases, hepatic fibrosis, and cystic‐fibrotic kidney disease. Brain phenotypes frequently manifest, including encephalocele, holoprosencephaly, microcephaly, polymicrogyria, heterotopia, intracerebral cysts, hippocampal dysgenesis, corpus callosum agenesis, hydrocephalus, cerebellar hypoplasia, cognitive deficits such as ID/DD, and ASD (Youn & Han, 2018). Regarding neuropsychiatric disorders, it has actually been suggested that primary cilia represent a conserved cellular structure at which the effects of diverse neuropsychiatric risk genes converge (Marley & von Zastrow, 2012).

Biallelic loss of function mutations in *TCTEX1D2* cause the ciliopathy Short rib thoracic dysplasia, which is characterized by a constricted thoracic cage, short ribs, and shortened tubular bones (Gholkar et al., 2015; Mukhopadhyay, 2015; Schmidts et al., 2015). Nonskeletal involvement can include cleft lip/palate, as well as anomalies of major organs. Our patient did not display most of these features. However, he had restricted thoracic cage leading to restrictive respiratory disease.

TCTEX1D2 has broad expression in the brain. In mice, Tctex1d2 has been found to have a physiological function related to insulin‐stimulated glucose uptake through the regulation of Glut4 receptor translocation (Shimoda, Okada, Yamada, Pessin, & Yamada, 2015). Glut4 receptor translocation is implicated in hippocampus‐dependent memory, as well as in the integration of hormonal and nutritional cues to mediate the actions of insulin on energy balance in the CNS (Pearson‐Leary & McNay, 2016; Ren, Lu, McGraw, & Accili, 2015). It is therefore possible that TCTEX1D2 may influence cognitive function, as well as the nutritional problems with “failure to thrive” seen in our patient and others with 3q29 deletion (Glassford et al., 2016). Altogether, supporting evidence suggest that impaired TCTEX1D2 expression and function are consistent with some features seen in our patient, but further experimental work too confirm that the variant affects splicing is warranted before we can suggest that *TCTEX1D2* variants may have a potential role in ASD.

Patient 2 carried a hemizygous predicted high‐risk variant in *DLG1* (rs1134986). The gene has not been connected to a Mendelian disease, as of today. DLG1 belongs to a family of scaffolding proteins, the membrane‐associated guanylate kinases (MAGUKs), that are highly enriched in the postsynaptic density of synapses and play an important role in organizing protein complexes necessary for synaptic development and plasticity (Gupta, Uner, Nayak, Grant, & Kalb, 2018). DLG1 regulates synaptic delivery of α‐amino‐3‐hydroxy‐5‐methyl‐4‐isoxazolepropionic acid (AMPA) receptors in hippocampal neurons (Nakagawa et al., 2004). It has been suggested that DLG1 may be important for the pruning process of neuron branches that occurs during adolescence and that alterations in this early brain remodeling mechanism may be the basis for the altered neural connectivity and functioning that can result in schizophrenia later in life (Cocchi, Drago, & Serretti, 2016).

*DLG1* has previously been associated with schizophrenia (Sato, Shimazu, Yamamoto, & Nishikawa, 2008; Toyooka et al., 2002; Uezato et al., 2012, 2015, 2017). The variant found in our patient was identified in a study of 234 unrelated cases with schizophrenia and 272 unrelated controls from the United Kingdom (Carroll et al., 2011). The allele frequency was >1% and did not differ between patients and controls (Carroll et al., 2011). This finding, as well as the presence of the allele in our patient´s healthy mother, indicates that the variant is not associated with schizophrenia when present in the heterozygote state. However, the effect of the variant in hemi‐ or homozygous states should be explored in greater depth. None of the patients carried the intronic variant rs3915512, which has previously been associated with reduced cortical expression of a splicing variant in DLG1 in patients with nonearly‐onset schizophrenia (Uezato et al., 2015), and with neurocognitive function in schizophrenia in general (Xu et al., 2018). Neither carried rs9843659, which has been associated with schizophrenia in male patients in a recessive model (Uezato et al., 2012).

Patient 2 also carried a predicted moderate‐risk variant (rs939885) in *SLC51A*. SLC51A is an essential component of the Ost‐alpha/Ost‐beta complex, a heterodimer that acts as an organic solute transporter of the sulfated neurosteroids pregnenolone sulfate (PREGS) and dehydroepiandrosterone sulfate (DHEAS) (Grube et al., 2018). Ost‐alpha deficient mice exhibited changes in serum DHEA and DHEAS levels, and in tissue distribution of administered DHEAS (Fang et al., 2010). In the human brain, the Ost‐alpha/Ost‐beta transporter is expressed in Purkinje cells and hippocampal neurons (Fang et al., 2010). After delivery to their target sites, they act as endogenous neurotransmitters or modulators on γ‐aminobutyric acid (GABA), particularly type A (GABA_A_), N‐methyl‐aspartate (NMDA) glutamate, serotonin (5‐HT3), and alpha‐1 receptors (Akwa, Ladurelle, Covey, & Baulieu, 2001; Smith, Gibbs, & Farb, 2014; Tuem & Atey, 2017; Whittaker, Gibbs, & Farb, 2008). PREGS may also act as a negative allosteric modulator of the AMPA, kainate, and glycine receptors, potassium channels, and voltage‐gated sodium channels; it may also inhibit voltage‐gated calcium channels and interact with nicotinic cholinergic receptors and sigma receptors (Rajagopal, Soni, & Meltzer, 2018). In this way, they exert potent effects on neuronal excitability and neurotransmitter receptor function. Modeling of the variant was not possible as no crystal structure is available, but the valine to isoleucine change is generally believed to be a conservative amino acid change. We suspect therefore that the present amino acid substitution might be too minor to lead to the functional impairment of enzyme activity.

Roles for PREGS and DHEAS in the etiopathogenesis of schizophrenia have been suggested (Vuksan‐Cusa, Sagud, & Rados, 2016; Wong, Sze, Chang, Lee, & Zhang, 2015). Blood DHEA and DHEAS levels of schizophrenia patients and healthy subjects have been found to differ across studies, ranging from normal to low and to high levels. Although several randomized, double‐blind, placebo‐controlled clinical trials have been conducted with DHEA(S) for the augmentation treatment of schizophrenia, their results have been conflicting (Vuksan‐Cusa et al., 2016). In a dopamine transporter knockout mouse model of schizophrenia, acute treatment with PREGS was able to rescue behavioral anomalies through the NMDA receptor‐mediated AKT/GSK3β signaling pathway (Wong et al., 2015). The current body of evidence supports further research with PREGS as a cognitive enhancer, as well as for treating negative symptoms in schizophrenia and related disorders (Rajagopal et al., 2018). The role of the identified risk variant in *SLC51A* in schizophrenia should be explored further.

Some of the risk variants we identified were also present as homozygous in first‐degree relatives. We have no indication that these variants are relevant to the patients’ phenotype.

In addition to genetic risk variants, haploinsufficiency of dose‐response genes may contribute to a certain phenotype (Costain et al., 2019). Our patients are hemizygous for TFRC. The receptor is primarily involved in iron homeostasis by regulating cellular iron uptake, and haploinsufficiency of TFRC is known to influence iron homeostasis with hematological abnormalities similar to those of our patients (Levy, Jin, Fujiwara, Kuo, & Andrews, 1999; Matak et al., 2016). Whether disturbed iron homeostasis and hematological abnormalities may be related to the chronic fatigue experienced by our patients is currently unknown.

The small sample size consisting of only two families is an important limitation of our study. The patients have different population background, which potentially may impact psychiatrically relevant phenotypes. However, the 3q29 deletion is known to be associated with ASD and schizophrenia both in Caucasian and Asian populations (Glassford et al., 2016; Kushima et al., 2018).

In conclusion, our in‐depth characterization of two adult carriers of the 3q29 deletion have broadened the phenotypic spectrum of this syndrome and shown that the deletion carriers may undergo a developmental phenotypic transition. The chronic pain and fatigue experienced by both patients underscores the need for regular clinical follow‐up and support. We have identified some risk variants in the remaining hemizygous allele that should be explored in greater depth regarding a possible role in neuropsychiatric disorders in a broader patient population. Our study points at genes involved in cilia function and neurosteroid transport, in addition to neural connectivity and excitability, neurotransmitter receptor function, and synaptic plasticity. Improved knowledge of the functional roles of genes and gene variants in the 3q29 chromosomal region may aid us in devising individually targeted, personalized interventions in 3q29 deletion carriers and contribute to our understanding of serious neuropsychiatric disorders such as autism and schizophrenia in general.

## CONFLICT OF INTEREST

The authors have no conflict of interest to declare.

## References

[mgg3889-bib-0001] Akwa, Y. , Ladurelle, N. , Covey, D. F. , & Baulieu, E. E. (2001). The synthetic enantiomer of pregnenolone sulfate is very active on memory in rats and mice, even more so than its physiological neurosteroid counterpart: Distinct mechanisms? Proceedings of the National Academy of Sciences USA, 98(24), 14033–14037. 10.1073/pnas.241503698 PMC61162 11717462

[mgg3889-bib-0002] Cai, H. , Cao, T. , Zhou, X. , & Yao, J. K. (2018). Neurosteroids in schizophrenia: Pathogenic and therapeutic implications. Frontiers in Psychiatry, 9, 73 10.3389/fpsyt.2018.00073 29568275 PMC5852066

[mgg3889-bib-0003] Carroll, L. S. , Williams, H. J. , Walters, J. , Kirov, G. , O'Donovan, M. C. , & Owen, M. J. (2011). Mutation screening of the 3q29 microdeletion syndrome candidate genes DLG1 and PAK2 in schizophrenia. American Journal of Medical Genetics. Part B, Neuropsychiatric Genetics: The Official Publication of the International Society of Psychiatric Genetics, 156B(7), 844–849. 10.1002/ajmg.b.31231 21850710

[mgg3889-bib-0004] Chénier, S. , Yoon, G. , Argiropoulos, B. , Lauzon, J. , Laframboise, R. , Ahn, J. , … Stavropoulos, D. J. (2014). CHD2 haploinsufficiency is associated with developmental delay, intellectual disability, epilepsy and neurobehavioural problems. Journal of Neurodevelopmental Disorders, 6(1), 9 10.1186/1866-1955-6-9 24834135 PMC4022362

[mgg3889-bib-0005] Cingolani, P. , Platts, A. , Wang, L. L. , Coon, M. , Nguyen, T. , Wang, L. , … Ruden, D. M. (2012). A program for annotating and predicting the effects of single nucleotide polymorphisms, SnpEff: SNPs in the genome of *Drosophila melanogaster* strain w1118; iso‐2; iso‐3. Fly (Austin), 6(2), 80–92. 10.4161/fly.19695 22728672 PMC3679285

[mgg3889-bib-0006] Città, S. , Buono, S. , Greco, D. , Barone, C. , Alfei, E. , Bulgheroni, S. , … Romano, C. (2013). 3q29 microdeletion syndrome: Cognitive and behavioral phenotype in four patients. American Journal of Medical Genetics Part A, 161A(12), 3018–3022. 10.1002/ajmg.a.36142 24214349

[mgg3889-bib-0007] Clayton‐Smith, J. , Giblin, C. , Smith, R. A. , Dunn, C. , & Willatt, L. (2010). Familial 3q29 microdeletion syndrome providing further evidence of involvement of the 3q29 region in bipolar disorder. Clinical Dysmorphology, 19(3), 128–132. 10.1097/MCD.0b013e32833a1e3c 20453639

[mgg3889-bib-0008] Cocchi, E. , Drago, A. , & Serretti, A. (2016). Hippocampal pruning as a new theory of schizophrenia etiopathogenesis. Molecular Neurobiology, 53(3), 2065–2081. 10.1007/s12035-015-9174-6 25902861

[mgg3889-bib-0009] Costain, G. , Walker, S. , Argiropoulos, B. , Baribeau, D. A. , Bassett, A. S. , Boot, E. , … Scherer, S. W. (2019). Rare copy number variations affecting the synaptic gene DMXL2 in neurodevelopmental disorders. Journal of Neurodevelopmental Disorders, 11(1), 3 10.1186/s11689-019-9263-3 30732576 PMC6366120

[mgg3889-bib-0010] Cox, D. M. , & Butler, M. G. (2015). A clinical case report and literature review of the 3q29 microdeletion syndrome. Clinical Dysmorphology, 24(3), 89–94. 10.1097/MCD.0000000000000077 25714563 PMC5125389

[mgg3889-bib-0011] Davis, L. K. , Meyer, K. J. , Rudd, D. S. , Librant, A. L. , Epping, E. A. , Sheffield, V. C. , & Wassink, T. H. (2009). Novel copy number variants in children with autism and additional developmental anomalies. Journal of Neurodevelopmental Disorders, 1(4), 292–301. 10.1007/s11689-009-9013-z 21547721 PMC3164008

[mgg3889-bib-0012] Fang, F. , Christian, W. V. , Gorman, S. G. , Cui, M. , Huang, J. , Tieu, K. , & Ballatori, N. (2010). Neurosteroid transport by the organic solute transporter OSTalpha‐OSTbeta. Journal of Neurochemistry, 115(1), 220–233. 10.1111/j.1471-4159.2010.06920.x 20649839 PMC2939961

[mgg3889-bib-0013] Gholkar, A. A. , Senese, S. , Lo, Y.‐C. , Capri, J. , Deardorff, W. J. , Dharmarajan, H. , … Torres, J. Z. (2015). Tctex1d2 associates with short‐rib polydactyly syndrome proteins and is required for ciliogenesis. Cell Cycle, 14(7), 1116–1125. 10.4161/15384101.2014.985066 25830415 PMC4614626

[mgg3889-bib-0014] Glassford, M. R. , Rosenfeld, J. A. , Freedman, A. A. , Zwick, M. E. & Mulle, J. G. (2016). Novel features of 3q29 deletion syndrome: Results from the 3q29 registry. American Journal of Medical Genetics. Part A, 170A(4), 999–1006. 10.1002/ajmg.a.37537 26738761 PMC4849199

[mgg3889-bib-0015] Gothelf, D. , Eliez, S. , Thompson, T. , Hinard, C. , Penniman, L. , Feinstein, C. , … Reiss, A. L. (2005). COMT genotype predicts longitudinal cognitive decline and psychosis in 22q11.2 deletion syndrome. Nature Neuroscience, 8(11), 1500–1502. 10.1038/nn1572 16234808

[mgg3889-bib-0016] Grayton, H. M. , Fernandes, C. , Rujescu, D. , & Collier, D. A. (2012). Copy number variations in neurodevelopmental disorders. Progress in Neurobiology, 99(1), 81–91. 10.1016/j.pneurobio.2012.07.005 22813947

[mgg3889-bib-0017] Grube, M. , Hagen, P. , & Jedlitschky, G. (2018). Neurosteroid Transport in the Brain: Role of ABC and SLC Transporters. Frontiers in Pharmacology, 9, 354 10.3389/fphar.2018.00354 29695968 PMC5904994

[mgg3889-bib-0018] Guipponi, M. , Santoni, F. , Schneider, M. , Gehrig, C. , Bustillo, X. B. , Kates, W. R. , … Antonarakis, S. E. (2017). No evidence for the presence of genetic variants predisposing to psychotic disorders on the non‐deleted 22q11.2 allele of VCFS patients. Translational Psychiatry, 7, e1039 10.1038/tp.2016.258. https://www.nature.com/articles/tp2016258#supplementary-information 28221368 PMC5438018

[mgg3889-bib-0019] Gupta, P. , Uner, O. E. , Nayak, S. , Grant, G. R. , & Kalb, R. G. (2018). SAP97 regulates behavior and expression of schizophrenia risk enriched gene sets in mouse hippocampus. PLoS ONE, 13(7), e0200477 10.1371/journal.pone.0200477 29995933 PMC6040763

[mgg3889-bib-0020] Kirov, G. (2015). CNVs in neuropsychiatric disorders. Human Molecular Genetics, 24(R1), R45–49. 10.1093/hmg/ddv253 26130694

[mgg3889-bib-0021] Kushima, I. , Aleksic, B. , Nakatochi, M. , Shimamura, T. , Okada, T. , Uno, Y. , … Ozaki, N. (2018). Comparative analyses of copy‐number variation in autism spectrum disorder and schizophrenia reveal etiological overlap and biological insights. Cell Reports, 24(11), 2838–2856. 10.1016/j.celrep.2018.08.022 30208311

[mgg3889-bib-0022] Leiknes, K. A. , Malt, U. F. , Malt, E. A. , & Leganger, S. (2006). M.I.N.I. International neuropsychiatric interview, Norwegian version (pp. 27). Jacksonville, FL: Medical Outcomes Systems Inc.

[mgg3889-bib-0023] Levy, J. E. , Jin, O. , Fujiwara, Y. , Kuo, F. , & Andrews, N. C. (1999). Transferrin receptor is necessary for development of erythrocytes and the nervous system. Nature Genetics, 21(4), 396–399. 10.1038/7727 10192390

[mgg3889-bib-0024] Liu, K. E. , Lei, R. , Li, Q. , Wang, X.‐X. , Wu, Q. , An, P. , … Li, H. (2016). Transferrin receptor controls AMPA receptor trafficking efficiency and synaptic plasticity. Scientific Reports, 6, 21019 10.1038/srep21019 26880306 PMC4754636

[mgg3889-bib-0025] Lord, C. , Rutter, M. , Goode, S. , Heemsbergen, J. , Jordan, H. , Mawhood, L. , & Schopler, E. (1989). Autism diagnostic observation schedule: A standardized observation of communicative and social behavior. Journal of Autism and Developmental Disorders, 19(2), 185–212. 2745388 10.1007/BF02211841

[mgg3889-bib-0026] Lord, C. , Rutter, M. , & Le Couteur, A. (1994). Autism diagnostic interview‐revised: A revised version of a diagnostic interview for caregivers of individuals with possible pervasive developmental disorders. Journal of Autism and Developmental Disorders, 24(5), 659–685. 10.1007/BF02172145 7814313

[mgg3889-bib-0027] Marley, A. , & von Zastrow, M. (2012). A simple cell‐based assay reveals that diverse neuropsychiatric risk genes converge on primary cilia. PLoS ONE, 7(10), e46647 10.1371/journal.pone.0046647 23056384 PMC3463515

[mgg3889-bib-0028] Matak, P. , Matak, A. , Moustafa, S. , Aryal, D. K. , Benner, E. J. , Wetsel, W. , & Andrews, N. C. (2016). Disrupted iron homeostasis causes dopaminergic neurodegeneration in mice. Proceedings of the National Academy of Sciences USA, 113(13), 3428–3435. 10.1073/pnas.1519473113 PMC4822577 26929359

[mgg3889-bib-0029] McKenna, A. , Hanna, M. , Banks, E. , Sivachenko, A. , Cibulskis, K. , Kernytsky, A. , … DePristo, M. A. (2010). The genome analysis toolkit: A MapReduce framework for analyzing next‐generation DNA sequencing data. Genome Research, 20(9), 1297–1303. 10.1101/gr.107524.110 20644199 PMC2928508

[mgg3889-bib-0030] Mukhopadhyay, S. (2015). TCTEX1D2, a potential link to skeletal ciliopathies. Cell Cycle, 14(3), 293–294. 10.1080/15384101.2015.1006548 25590661 PMC4353232

[mgg3889-bib-0031] Mulle, J. G. (2015). The 3q29 deletion confers >40‐fold increase in risk for schizophrenia. Molecular Psychiatry, 20(9), 1028–1029. 10.1038/mp.2015.76 26055425 PMC4546529

[mgg3889-bib-0032] Mulle, J. G. , Dodd, A. F. , McGrath, J. A. , Wolyniec, P. S. , Mitchell, A. A. , Shetty, A. C. , … Warren, S. T. (2010). Microdeletions of 3q29 confer high risk for schizophrenia. American Journal of Human Genetics, 87(2), 229–236. 10.1016/j.ajhg.2010.07.013 20691406 PMC2917706

[mgg3889-bib-0033] Nakagawa, T. , Futai, K. , Lashuel, H. A. , Lo, I. , Okamoto, K. , Walz, T. , … Sheng, M. (2004). Quaternary structure, protein dynamics, and synaptic function of SAP97 controlled by L27 domain interactions. Neuron, 44(3), 453–467. 10.1016/j.neuron.2004.10.012 15504326

[mgg3889-bib-0034] Pearson‐Leary, J. , & McNay, E. C. (2016). Novel roles for the insulin‐regulated glucose transporter‐4 in hippocampally dependent memory. Journal of Neuroscience, 36(47), 11851–11864. 10.1523/JNEUROSCI.1700-16.2016 27881773 PMC5125244

[mgg3889-bib-0035] Quintero‐Rivera, F. , Sharifi‐Hannauer, P. , & Martinez‐Agosto, J. A. (2010). Autistic and psychiatric findings associated with the 3q29 microdeletion syndrome: Case report and review. American Journal of Medical Genetics. Part A, 152A(10), 2459–2467. 10.1002/ajmg.a.33573 20830797

[mgg3889-bib-0036] Rajagopal, L. , Soni, D. , & Meltzer, H. Y. (2018). Neurosteroid pregnenolone sulfate, alone, and as augmentation of lurasidone or tandospirone, rescues phencyclidine‐induced deficits in cognitive function and social interaction. Behavioral Brain Research, 350, 31–43. 10.1016/j.bbr.2018.05.005 29763637

[mgg3889-bib-0037] Raven, J. (2000). The Raven's progressive matrices: Change and stability over culture and time. Cognitive Psychology, 41(1), 1–48. 10.1006/cogp.1999.0735 10945921

[mgg3889-bib-0038] Ren, H. , Lu, T. Y. , McGraw, T. E. , & Accili, D. (2015). Anorexia and impaired glucose metabolism in mice with hypothalamic ablation of Glut4 neurons. Diabetes, 64(2), 405–417. 10.2337/db14-0752 25187366 PMC4303970

[mgg3889-bib-0039] Sagar, A. , Bishop, J. R. , Tessman, D. C. , Guter, S. , Martin, C. L. , & Cook, E. H. (2013). Co‐occurrence of autism, childhood psychosis, and intellectual disability associated with a de novo 3q29 microdeletion. American Journal of Medical Genetics. Part A, 161A(4), 845–849. 10.1002/ajmg.a.35754 23443968 PMC3685481

[mgg3889-bib-0040] Sato, J. , Shimazu, D. , Yamamoto, N. , & Nishikawa, T. (2008). An association analysis of synapse‐associated protein 97 (SAP97) gene in schizophrenia. Journal of Neural Transmission, 115(9), 1355–1365. 10.1007/s00702-008-0085-9 18665322

[mgg3889-bib-0041] Schmidts, M. , Hou, Y. , Cortés, C. R. , Mans, D. A. , Huber, C. , Boldt, K. , … Witman, G. B. (2015). TCTEX1D2 mutations underlie Jeune asphyxiating thoracic dystrophy with impaired retrograde intraflagellar transport. Nature Communications, 6, 7074 10.1038/ncomms8074 PMC4468853 26044572

[mgg3889-bib-0042] Shimoda, Y. , Okada, S. , Yamada, E. , Pessin, J. E. , & Yamada, M. (2015). Tctex1d2 Is a negative regulator of GLUT4 translocation and glucose uptake. Endocrinology, 156(10), 3548–3558. 10.1210/en.2015-1120 26200093 PMC5398638

[mgg3889-bib-0043] Smith, C. C. , Gibbs, T. T. , & Farb, D. H. (2014). Pregnenolone sulfate as a modulator of synaptic plasticity. Psychopharmacology (Berl), 231(17), 3537–3556. 10.1007/s00213-014-3643-x 24997854 PMC4625978

[mgg3889-bib-0044] Sparrow, S. , Balla, D. A. , & Cicchetti, D. (1984). Vineland adaptive behavior scales. Circle Pines, MN: American Guidance Services.

[mgg3889-bib-0045] Sparrow, S. S. , Cicchetti, D. V. , & Balla, D. A. (2005). Vineland adaptive behavior scales. 2nd edn. Circle Pines, MN: American Guidance Services.

[mgg3889-bib-0046] Swillen, A. , & McDonald‐McGinn, D. (2015). Developmental trajectories in 22q11.2 deletion. American Journal of Medical Genetics Part C: Seminars in Medical Genetics, 169(2), 172–181. 10.1002/ajmg.c.31435 PMC5061035 25989227

[mgg3889-bib-0047] Torres, F. , Barbosa, M. , & Maciel, P. (2016). Recurrent copy number variations as risk factors for neurodevelopmental disorders: Critical overview and analysis of clinical implications. Journal of Medical Genetics, 53(2), 73–90. 10.1136/jmedgenet-2015-103366 26502893

[mgg3889-bib-0048] Toyooka, K. , Iritani, S. , Makifuchi, T. , Shirakawa, O. , Kitamura, N. , Maeda, K. , … Nawa, H. (2002). Selective reduction of a PDZ protein, SAP‐97, in the prefrontal cortex of patients with chronic schizophrenia. Journal of Neurochemistry, 83(4), 797–806. 10.1046/j.1471-4159.2002.01181.x 12421351

[mgg3889-bib-0049] Tuem, K. B. , & Atey, T. M. (2017). Neuroactive steroids: Receptor interactions and responses. Front Neurol, 8, 442 10.3389/fneur.2017.00442 28894435 PMC5581316

[mgg3889-bib-0050] Uezato, A. , Kimura‐Sato, J. , Yamamoto, N. , Iijima, Y. , Kunugi, H. , & Nishikawa, T. (2012). Further evidence for a male‐selective genetic association of synapse‐associated protein 97 (SAP97) gene with schizophrenia. Behavioral and Brain Functions, 8, 2 10.1186/1744-9081-8-2 22225629 PMC3275478

[mgg3889-bib-0051] Uezato, A. , Yamamoto, N. , Iwayama, Y. , Hiraoka, S. , Hiraaki, E. , Umino, A. , … Nishikawa, T. (2015). Reduced cortical expression of a newly identified splicing variant of the DLG1 gene in patients with early‐onset schizophrenia. Translational Psychiatry, 5, e654 10.1038/tp.2015.154 26440542 PMC4930131

[mgg3889-bib-0052] Uezato, A. , Yamamoto, N. , Jitoku, D. , Haramo, E. , Hiraaki, E. , Iwayama, Y. , … Nishikawa, T. (2017). Genetic and molecular risk factors within the newly identified primate‐specific exon of the SAP97/DLG1 gene in the 3q29 schizophrenia‐associated locus. American Journal of Medical Genetics. Part B, Neuropsychiatric Genetics: The Official Publication of the International Society of Psychiatric Genetics., 174(8), 798–807. 10.1002/ajmg.b.32595 28990294

[mgg3889-bib-0053] Vuksan‐Cusa, B. , Sagud, M. , & Rados, I. (2016). The role of dehydroepiandrosterone (DHEA) in schizophrenia. Psychiatria Danubina, 28(1), 30–33. 26938818

[mgg3889-bib-0054] Wechsler, D. , Coalson, D. L. , & Raiford, S. E. (2008). Wechsler adult intelligence scale‐fourth‐WAIS‐IV. San Antonio, TX: Pearson.

[mgg3889-bib-0055] Whittaker, M. T. , Gibbs, T. T. , & Farb, D. H. (2008). Pregnenolone sulfate induces NMDA receptor dependent release of dopamine from synaptic terminals in the striatum. Journal of Neurochemistry, 107(2), 510–521. 10.1111/j.1471-4159.2008.05627.x 18710414 PMC2752276

[mgg3889-bib-0056] Willatt, L. , Cox, J. , Barber, J. , Cabanas, E. D. , Collins, A. , Donnai, D. , … Raymond, F. L. (2005). 3q29 microdeletion syndrome: Clinical and molecular characterization of a new syndrome. American Journal of Human Genetics, 77(1), 154–160. 10.1086/431653 15918153 PMC1226188

[mgg3889-bib-0057] Wong, P. , Sze, Y. , Chang, C. C. , Lee, J. , & Zhang, X. (2015). Pregnenolone sulfate normalizes schizophrenia‐like behaviors in dopamine transporter knockout mice through the AKT/GSK3beta pathway. Translational Psychiatry, 5, e528 10.1038/tp.2015.21 25781227 PMC4354351

[mgg3889-bib-0058] Xu, X. , Liang, C. , Lv, D. , Yin, J. , Luo, X. , Fu, J. , … Li, K. (2018). Association of the synapse‐associated protein 97 (SAP97) gene polymorphism with neurocognitive function in schizophrenic patients. Frontiers in Psychiatry, 9, 458 10.3389/fpsyt.2018.00458 30319465 PMC6169480

[mgg3889-bib-0059] Youn, Y. H. , & Han, Y. G. (2018). Primary cilia in brain development and diseases. American Journal of Pathology, 188(1), 11–22. 10.1016/j.ajpath.2017.08.031 29030052 PMC5745523

[mgg3889-bib-0060] Zarrei, M. , MacDonald, J. R. , Merico, D. , & Scherer, S. W. (2015). A copy number variation map of the human genome. Nature Reviews Genetics, 16(3), 172–183. 10.1038/nrg3871 25645873

[mgg3889-bib-0061] Zhang, Z. , Li, H. , Chen, L. , Lu, X. , Zhang, J. , Xu, P. , … Wu, G. (2011). Molecular basis for the recognition of adenomatous polyposis coli by the Discs Large 1 protein. PLoS ONE, 6(8), e23507 10.1371/journal.pone.0023507 21858148 PMC3157396

